# Subthreshold eosinophilia during food impaction may mask eosinophilic esophagitis: A pediatric case series

**DOI:** 10.1002/jpr3.70114

**Published:** 2025-11-14

**Authors:** Mark Mahon, Amanda Muir

**Affiliations:** ^1^ Division of Gastroenterology, Hepatology, and Nutrition Children's Hospital of Philadelphia Philadelphia Pennsylvania USA; ^2^ Perelman School of Medicine at the University of Pennsylvania Philadelphia Pennsylvania USA

**Keywords:** atopic, degranulated, dysphagia, esophagus, mucosa

## Abstract

To describe the clinical scenario and need for longitudinal follow‐up of pediatric patients presenting with esophageal food impactions (EFIs) regardless of esophageal biopsy eosinophil count. A retrospective chart review was conducted on patients with EFI who underwent endoscopic evaluation from August 2022 to August 2024. Patients demonstrating eosinophils per high‐power field (HPF) between 1 and 14 were identified, and longitudinal assessment evaluated for potential eosinophilic esophagitis (EoE) diagnosis. Four patients presented with EFI and sub‐diagnostic eosinophilic counts (1–14 eosinophils/HPF) were assessed longitudinally. In all cases, repeat endoscopy showed an increase in eosinophils, confirming EoE (eosinophils ≥ 15/HPF). EFI is a potential presenting sign of EoE, but initial biopsy results during EFI often show sub‐diagnostic eosinophilic counts. Our findings underscore the need for adequate biopsy procurement at the time of EFI, longitudinal follow‐up, and repeat endoscopy for accurate EoE diagnosis during EFI.

## Abstract (highlights)

**What is Known:**

- EoE diagnosis is a clinicopathologic diagnosis with symptoms of esophageal dysfunction and ≥15 eosinophilic cells per HPF.
- Eosinophils are extremely sensitive to their environment and undergo rapid degranulation during times of mucosal stress.
- Esophageal food bolus impaction is a frequent presenting sign in EoE.

**What is New:**

- A subgroup of patients during esophageal food impactions has been identified with lower than diagnostic thresholds for EoE on eosinophil per HPF count.
- Repeat mucosal evaluation before empiric treatment, when esophageal mucosa has recovered, can be confirmatory of a diagnosis of EoE.
- Follow‐up in patients with food impaction, regardless of eosinophil count, is essential.

## 1 INTRODUCTION

EFIs are an established presentation of EoE. Chronic inflammation leads to fibrostenosis, leading to difficulty with esophageal propagation of the food bolus. Both the inflammatory burden and esophageal stiffness result in EFI and the need for endoscopic intervention. Despite the fact that EoE is a major cause of EFI, only 80% of cases presenting with EFI have routine esophageal biopsies.^1^ The diagnostic yield for EoE increases when greater than routine biopsies are obtained, that is, obtaining at least six and sampling multiple levels.^2^

One issue that arises during EFI is that the biopsies obtained at the time of the emergency procedure do not provide the requisite ≥15 eosinophils per HPF to make a diagnosis of EoE, leading to diagnostic and therapeutic dilemmas. We aim to highlight in this case series four clinical cases where diagnostic eosinophil/HPF was subthreshold during index upper endoscopy for EFI.

Herein, we aim to highlight the importance of comprehensive evaluation of the esophagus during an EFI, the need for thorough endoscopic evaluation, and the importance of longitudinal follow‐up regardless of eosinophil counts during food impaction.

## 2 METHODS

### 2.1 Ethics statement

Our institutional review board (IRB) was approved by The Children's Hospital of Philadelphia Institutional Review Board.

### 2.2 Study design

Under our approved IRB (24‐021921), we performed a retrospective chart review to identify patients who had an endoscopic evaluation at the time of an EFI. Endoscopic reports were reviewed to identify the gross pathological findings, the number of esophageal biopsies taken, and the location stratified into upper, mid‐, and distal esophagus. Based on histological analysis, this was able to stratify the patients as diagnostic of EoE (≥15 eosinophils per HPF)^3^ or those with sub‐diagnostic thresholds between 1 and 14 eosinophils/HPF.

## 3 RESULTS

### 3.1 Patient characteristics

Over a 2‐year period, between August 2022 and August 2024, a total of seven EFI patients were identified who, after endoscopy by a pediatric gastroenterologist to remove food bolus where biopsies were procured, were found to have between 1 and 14 eosinophils/HPF on histology. Three of these patients were lost to follow‐up, and therefore longitudinal data on clinical symptomatology and re‐evaluation with a repeat upper endoscopy were not available.

### 3.2 Patient A

A 17‐year‐old male with a history of immunoglobulin E (IgE)‐mediated peanut allergy and eczema presented with acute dysphagia after consuming beef. He reported that he felt the food item get “stuck in his throat” and thereafter was unable to tolerate liquid by mouth or his own secretions. He had a fluoroscopic esophagram performed, which identified a food impaction at the mid‐esophagus with complete obstruction. He underwent an upper endoscopy where the food item was identified at the mid‐esophagus and removed with a rat tooth forceps. The gross description of his esophageal mucosa was noted by edema, furrows, and exudates involving the entire esophagus; giving an Eosinophilic Esophagitis Endoscopic Reference Score (EREFS) of 4. He had three esophageal biopsy specimens obtained throughout the esophagus. The results of his biopsies revealed moderate esophagitis with full‐thickness basal cell hyperplasia associated with moderate intercellular edema. There was a mixed inflammatory infiltrate with neutrophils, lymphocytes, but only rare (<5) eosinophils. There was scant lamina propria, but this did have apparent fibrosis in a few fragments, which also contained degranulated eosinophils.

After resolution of his EFI, he was started on high‐dose proton‐pump inhibitor and re‐scoped 12 weeks later. This second endoscopy continued to show exudates and furrows only, with an EREFS of 2. At this repeat endoscopy, a total of six biopsies were obtained throughout the esophagus. The histology was confirmatory for EoE with 55 eosinophils/HPF with persistence of secondary features of epithelial basal cell hyperplasia and intercellular edema. After the addition of topical Budesonide slurry 2 mg daily, he entered histological remission.

### 3.3 Patient B

A 16‐year‐old male with a history of asthma presented with acute dysphagia after eating steak tacos. He reported symptoms for several months prior, which he described as “choking with eating.” He had an esophagram performed, which identified the food impaction with complete obstruction. He underwent an upper endoscopy, whereby the food item was removed with a suction cap and rat‐tooth forceps. After removal, the gross description was detailed as edematous and with furrows along the length of the esophagus, with an EREFS of 2. He had four esophageal biopsy specimens obtained. His histology showed only 10 eosinophils/HPF with noted basal cell layer hyperplasia and fibrosis on the lamina propria.

He was not started on any empiric treatment and established care with an outpatient gastroenterologist. He underwent repeat upper endoscopy 6 weeks later, where the appearance of a furrowed esophagus persisted (EREFS of 1), but now histology showed basal cell hyperplasia with 60 eosinophils/HPF. He was started on high‐dose PPI and is awaiting repeat endoscopy.

### 3.4 Patient C

A 5‐year‐old male with a prior history of choking and no other atopy presented with gagging, coughing, and drooling after eating oatmeal with raisins and almonds. At the time of presentation, he was unable to tolerate his secretions and had an esophagram, which showed an incomplete filling defect in the mid‐thoracic esophagus consistent with a partial obstruction. He underwent an upper endoscopy, which identified the almond in the mid‐esophagus, which was removed with a rat‐tooth forceps. The entire esophagus was congested, with localized erosions around the site of the EFI giving an EREFS of 1. He had eight esophageal biopsy specimens obtained. On histology, the squamous mucosa was inflamed but with only five eosinophils/HPF. There was evidence of basal cell layer hyperplasia and fibrosis of the lamina propria.

After the EFI, he reported ongoing dysphagia and established care with gastroenterology. He underwent repeat endoscopic evaluation 4 months later, without any interval treatment, and was found to have gross esophagitis noted by edema, furrows, and exudates along the length of the esophagus. EREFS was 3 at the time. His histology revealed persistence of the basal cell layer hyperplasia and fibrosis of the lamina propria, now with 40–50 eosinophils/HPF. He was started on high‐dose PPI and is awaiting repeat endoscopy.

### 3.5 Patient D

A 13‐year‐old male with an IgE‐mediated peanut allergy presented with dysphagia and emesis after eating steak and sweet potatoes. He did not report any dysphagia before this event. He underwent an esophagram, which identified an obstructing food bolus in the distal esophagus. During upper endoscopy, the food bolus was removed with rat‐tooth forceps, and the gross description of the esophagus was described with longitudinal furrows. The EREFS was 1. He had four biopsies obtained from the esophagus. His biopsies resulted in a histological description of esophagitis noted by mixed infiltration with neutrophils, but only three eosinophils/HPF. There was basal cell hyperplasia with intercellular edema, and although the lamina propria was scant, there was condensation suspicious for fibrosis.

After the endoscopy, he had resolution of his symptoms and was prescribed a proton pump inhibitor at a high dose of 40 mg twice daily. He underwent repeat endoscopy 4 months later, on this treatment regimen. This revealed worsening features of esophagitis, now with the addition of a ringed appearance (EREFS of 5), while passage of the regular pediatric endoscope led to a mucosal tear. His histology at repeat scope reached diagnostic thresholds with 15 eosinophils/HPF, and he was started on treatment with oral viscous budesonide slurry.

## 4 DISCUSSION

Herein, we describe the longitudinal clinical course of four patients, each of whom presented with EFI and biopsies demonstrating subclinical threshold eosinophil levels to make a definitive diagnosis of EoE. Subsequently, each patient received the diagnosis of EoE on a repeat endoscopy. This case series aims to highlight the importance of comprehensive evaluation of the esophagus during an EFI and the importance of Gastroenterology follow‐up for all patients with EFI.

EFIs can be a presenting sign of EoE.^1^ Because of coping mechanisms, patients may not be aware of chronic dysphagia before presenting with an EFI. It is known that children can have adaptive eating behaviors, such as excessive chewing or consumption of beverages with meals, to manage their chronic subconscious symptom of dysphagia.^4^ Between 50% and 75% of patients with food bolus impaction have the diagnosis of EoE.^1^, ^5^ However, it is also well established that management of an EFI is variable, with surgical providers often providing after‐hours care. Due to provider discrepancy, not every patient may receive standard esophageal biopsies,^1^ and therefore the percentage of patients with EoE is plausibly higher in this cohort.

One possible explanation for low eosinophil counts in the EFI population is inadequate biopsy practices during an EFI. The American College of Gastroenterology recently recommended obtaining at least six esophageal biopsy specimens from multiple levels to best evaluate the esophagus, due to the patchy distribution of the disease.^2^ In our cohort of four patients, all patients received esophageal biopsies. The number of biopsies obtained in the esophagus during an EFI varied from 3 to 8. While eight biopsies should have been sufficient, three out of four had less than the recommended six. This may represent some trepidation on the part of the endoscopist to biopsy the esophagus that was injured by the food impaction, and therefore inadequate sampling leading to a missed diagnosis.

Another explanation for low eosinophil counts in the impacted esophagus is eosinophil degranulation. Eosinophils are known to be sensitive immune cells, prone to self‐degranulation with exocytosis of eosinophilic granules during times of stress.^6^ Routine hematoxylin and eosin (H&E) stain is used to identify peak eosinophil/HPF counts of intact eosinophils. The detection of eosinophil secretory granules using eosinophil peroxidase (EPX) has been shown to correlate with eosinophil count on H&E staining in EoE patients, but such targeted stains are not routinely available in clinical practice. This has led to the identification of a subcategory of patients with high EPX staining but <15 eosinophils/HPF.^7^, ^8^, ^9^ The use of EPX staining may be able to highlight those additional degranulated eosinophils needed to reach diagnostic thresholds during times of mucosal injury or stress, such as an EFI.

With insufficient sampling and limited universal access to eosinophilic protein stains to aid in the diagnosis of EoE during an EFI, secondary histological features, EREFS, and atopic history are essential for evaluating EFI patients. Certain features seen in our patients, such as basal cell hyperplasia, intercellular edema (spongiosis), and condensation of the lamina propria, were present on histology. While these can be nonspecific findings, they may help to support a diagnosis. Similarly, the endoscopic appearance of the food impaction patients demonstrated edema and furrows, but the presence of a normal appearing endoscopy should not preclude the procurement of biopsies in the case of a food impaction.^10^, ^11^, ^12^ Finally, for patients presenting with EFI, the endoscopist must always consider the patient's atopic comorbidities. The majority of EoE patients have an atopic comorbidity and identifying the occurrence of food/seasonal allergies, asthma, and/or atopic dermatitis before an endoscopy can raise the suspicion for EoE.^13^, ^14^

**Figure 1:**
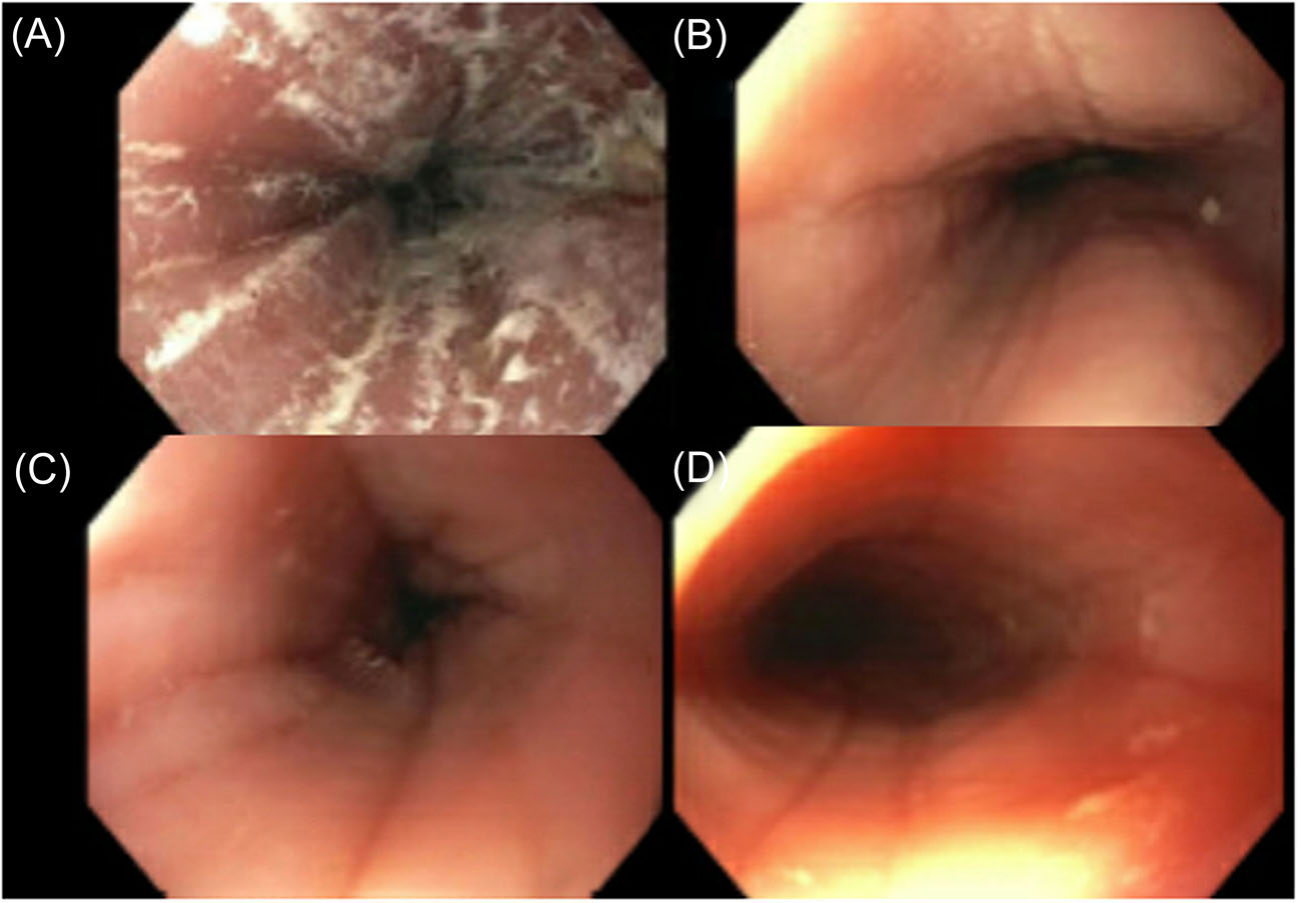
Endoscopic gross findings of esophagitis noted for patients (A–D), an index esophageal food impaction. Patient (A) demonstrates exudates, edema, and deep furrows. Patient (B) with edema and furrows. Patient (C) with edema and furrows, and Patient (D) with edema, furrows, and subtle rings.

Because EoE is a progressive fibrostenotic disease, early identification and diagnosis are essential. These missed opportunities for diagnosis delay treatment initiation. Missed opportunities to diagnose EoE not only arise during EFI when biopsies are not taken, but also when these patients with EFI are lost to follow‐up. The loss to follow‐up rate after EFI has been reported as high as 50%.^15^, ^16^ Our cohort of patients all received an index endoscopy at the time of EFI (Figure 1); however, three of the seven patients (42%) did not receive follow‐up. In fact, of our four patients who did, 100% received a diagnosis of EoE on follow‐up endoscopy.

## 5 CONCLUSION

Our case series highlights a subcategory of patients with eosinophilic/HPF thresholds below diagnostic criteria during an EFI, who ultimately were diagnosed with EoE on subsequent endoscopy when sufficient time was allowed for mucosal recovery from EFI. The endoscopist should therefore be aware that EoE is a pleomorphic disease, and not only could the esophagus appear visually normal during an EFI, but the histological definition may not be met with ≥15 eosinophils/HPF due to the mucosal stress leading to degranulation and/or lack of sufficient sampling. Our cases highlight the importance of adequate sampling when there is a clinical suspicion for EoE and to ensure timely follow‐up for all EFI patients.

## CONFLICT OF INTEREST STATEMENT

Dr. Amanda Muir serves on advisory boards for Sanofi/Regeneron, Uniquity, Takeda, and EsoCap. The remaining author declares no conflicts of interest.
